# Early eczema and the risk of childhood asthma: a prospective, population-based study

**DOI:** 10.1186/1471-2431-12-168

**Published:** 2012-10-24

**Authors:** Marit Saunes, Torbjørn Øien, Christian K Dotterud, Pål R Romundstad, Ola Storrø, Turid L Holmen, Roar Johnsen

**Affiliations:** 1 Department of Public Health and General Practice, Norwegian University of Science and Technology, Trondheim, Norway; 2 Department of Dermatology, St Olav Hospital, Trondheim University Hospital, Trondheim, Norway; 3 HUNT Research Centre, Department of Public Health and General Practice, Norwegian University of Science and Technology, Trondheim, Norway; 4 Department of Public Health and General Practice, Medical Research Centre (MTFS), NO-7489, Trondheim, Norway

**Keywords:** Eczema, Asthma, Child, Preschool, Cohort, Questionnaires

## Abstract

**Background:**

Severe eczema in young children is associated with an increased risk of developing asthma and rhino-conjunctivitis. In the general population, however, most cases of eczema are mild to moderate. In an unselected cohort, we studied the risk of current asthma and the co-existence of allergy-related diseases at 6 years of age among children with and without eczema at 2 years of age.

**Methods:**

Questionnaires assessing various environmental exposures and health variables were administered at 2 years of age. An identical health questionnaire was completed at 6 years of age. The clinical investigation of a random subsample ascertained eczema diagnoses, and missing data were handled by multiple imputation analyses.

**Results:**

The estimate for the association between eczema at 2 years and current asthma at 6 years was OR=1.80 (95% CI 1.10-2.96). Four of ten children with eczema at 6 years had the onset of eczema after the age of 2 years, but the co-existence of different allergy-related diseases at 6 years was higher among those with the onset of eczema before 2 years of age.

**Conclusions:**

Although most cases of eczema in the general population were mild to moderate, early eczema was associated with an increased risk of developing childhood asthma. These findings support the hypothesis of an atopic march in the general population.

**Trial registration:**

The Prevention of Allergy among Children in Trondheim study has been identified as ISRCTN28090297 in the international Current Controlled Trials database

## Background

The atopic march is a term used to describe the relationship between allergy-related diseases, starting with food allergy and eczema in early childhood, and the subsequent development of asthma and rhino-conjunctivitis. An association between eczema and asthma in childhood has been documented in several studies, and severe eczema is associated with an increased tendency to produce immunoglobulin E (IgE) and developing asthma [1-7]. The nature of the relationship between eczema and asthma has, however, been debated. Although these diseases share some genetic and environmental risk factors, it has been argued that eczema and asthma are unrelated and that the progression of eczema to asthma is due to the co-manifestation of eczema and wheezing early in life [3,6]. On the other hand, plausible biological pathways have been described, with eczema as the first step in a progressive atopic march leading to asthma and/or rhino-conjunctivitis [8,9]. It has also been argued that the atopic march is restricted to males only [1]. A defective skin barrier due to mutations in the gene encoding profilaggrin/filaggrin (FLG) is associated with increased trans-epidermal water-loss, dry skin, itching and eczema [10]. Studies on FLG mutations have not, however, found any association with FLG mutations and asthma/rhino-conjunctivitis in children who did not have eczema.

These findings suggest different phenotypes for both asthma and eczema [11-13].

Because previous studies have been based primarily on cross-sectional data, high-risk or hospitalised patients, population-based studies with prospective designs are warranted [3,5,6,14,15]. The aim of the present study was to prospectively investigate the association between a history of eczema at 2 years of age and current asthma at 6 years of age in a general population. We also aimed to determine the prevalence of allergy-related diseases at 6 years of age according to eczema status at 2 years of age.

## Methods

The Prevention of Allergy among Children in Trondheim (PACT) study is a comprehensive, controlled, primary intervention study of allergy-related diseases in Trondheim, central Norway. The study is described in detail elsewhere [16]. Briefly, the control cohort included pregnant women and parents with their young children at ages 6 weeks, 1 year, 2 years and 6 years attending ordinary scheduled appointments with GPs, midwives or health-workers. The inclusion of 2-year-olds started on September 1^st^ of 2000 and closed on March 31^st^ of 2005; the inclusion of 6-year-olds started on September 1^st^ of 2000 and closed on December 31^st^ of 2008. In the present study, children from the PACT control cohort with cross-sectional data at 2 years were followed-up at the age of 6 years. In addition, we used information on sensitisation from a random subsample of children evaluated clinically at 2 years of age.

### Study variables

Parents of children at age 2 (baseline) were given questionnaires concerning environmental exposures and the family history of different allergy-related diseases in the parents and siblings. A detailed questionnaire concerning the child’s health, with an emphasis on allergy-related diseases, was also administered. An identical health questionnaire was administered when the child was 6 years of age. The questions were adapted from the International Study of Asthma and Allergies in Childhood (ISAAC) study to fit both age groups.

Both questions “Has your child *ever* had eczema?” and “Has your child *ever* had an itchy rash which was coming and going for at least 6 months?” had to be answered positively to obtain the designation of “history of eczema” at 2 years of age. To identify children who had current eczema at 6 years of age, the two former questions were combined with “Has your child during the last 12 months used any kind of medication, ointment, cream, tablets or herbal medicines against eczema?” A history of asthma was denoted by a positive answer to the question “Has your child *ever* been diagnosed as having asthma by a doctor?” To obtain current asthma status, the former question was combined with a positive answer to “Has the child during the last 12 months used tablets, inhalation medications or other treatments for wheezing, tightness in the chest or asthma?”

The presence of wheezing was determined by a positive answer to both questions “Has your child *ever* had whistling in the chest?” and “Has your child *ever* had episodes of wheezing or tightness in the chest?”

Positive answers to the question “Has your child *ever* had hay fever, sneezing or itchy-watery eyes” defined a history of rhino-conjunctivitis.

A history of an allergy test was defined as an affirmative response to the question “Has your child *ever* been allergy tested by skin prick test or blood test?” A positive allergy test was present if the child’s parent(s) reported a positive reaction to at least one allergen.

“Atopy in the family” was registered as present if the child’s mother, father or any siblings had answered “yes” to one of several questions regarding asthma, eczema and allergic rhino-conjunctivitis.

The term “Homeowner” was used as a proxy for socioeconomic status and was based on the question “Do you/your family own your apartment/house”.

### The random subsample

From March 2001 to September 2002, a randomly sampled group of 720 pregnant women from the same control cohort described above were invited to attend a sub-study of PACT [17] . Their children were invited to a clinical examination at 2 years of age, including an assessment for eczema according to the United Kingdom Working Party (UKWP) diagnostic criteria. Disease severity was scored by the Scoring of Atopic Dermatitis (SCORAD) index and sensitisation was evaluated by standard prick test (SPT) and specific IgE (sIgE) measurements [14].

For SPT testing, the following standardised extracts from Soluprick® allergens (ALK Albello, Denmark) were used: mite (Dermatophagoides pteronyssinus), mould (Cladosporium herbarum), cat and dog dander, birch, timothy (grass) and mugwort pollens, hen’s egg white, codfish, hazelnut and peanut. For cow’s milk fresh skimmed milk was used. In addition, two positive histamine controls (Histamine 10mg/ml) and one diluents-negative control (NaCl) were applied on the volar surface of the child’s forearms. A mean wheal diameter of at least 3 mm larger than the negative control was considered a positive test.

Sera from venous blood samples were analysed for sIgE using assays testing for the same allergens as the SPTs. Venous sampling was attempted only once. The sIgE analyses were carried out in the immunology laboratory at St Olavs University Hospital, Trondheim, using the Immulite® 2000 Allergen-specific IgE system (Siemens Medical Solutions Diagnostics, Deerfield, IL, U.S.A.). A sIgE ≥0.35 kUL^-1^ was considered positive.

Sensitisation was defined as positive if the child either had a positive SPT or a positive sIgE.

### Statistics

Baseline characteristics as well as allergy-related diseases are described as prevalences with 95% confidence intervals (CI) for dichotomous variables.

Multivariable, logistic regression models were used to estimate the adjusted associations between a history of eczema at 2 years and current asthma at 6 years. Eczema and the age of eczema onset were used as explanatory variables. The age of eczema onset was divided in tertiles (0–3 months, 4–12 months, 13 months or older), and all other explanatory variables in the model were dichotomised. The possible confounding factors were identified by a priori knowledge, and in the final models, adjustments were made for sex, atopy in the family, wheezing, homeowner status and a smoking mother. Interaction on the multiplicative level was tested between eczema and sex and between eczema and wheezing using the likelihood ratio test. Children with a history of doctor-diagnosed asthma at age 2 years were excluded from the follow-up analyses. In addition, follow-up analyses excluding both children with doctor-diagnosed asthma and children who had ever experienced wheezing at age 2 years were performed.

### Missing data analysis

Due to the large amount of missing data, we used multiple imputations (MI) to assess the potential impact of missing data in the regression analyses. We assumed that data were missing at random given the results from the observed data, and we used chained equations (regression switching) with 50 sets of imputations to impute missing values as implemented in the STATA’s ICE command. The following predictor variables reported at 2 years were included in the imputation model: eczema, age in tertiles, siblings, atopy in the family, a cat in the household, symptoms of wheezing/whistling in the chest, symptoms of hay fever, history of hospitalisation for any allergy-related diseases, homeowner status, smoking mother/father and sensitisation. In addition, the outcome variable, current asthma at 6 years, was included. Because reported sensitisation was not considered missing at random, sensitisation-data from the subsample, where all of the randomly selected participants included were tested regardless of the presence of disease symptoms, were used as predictors in the ICE command. A multiplicative interaction term between eczema and sensitisation was also included in the predictive ICE command and tested in the final models. For each outcome variable, a separate MI dataset was created, and resulting estimates were combined by the MIM command in STATA. To study the association between a history of eczema at 2 years and current asthma at 6 years, logistic regression analyses were performed on the MI dataset.

In addition to the multiple imputation approach, we also performed analyses based on individuals with complete data.

All data were analysed using STATA for Windows (version 11, College Station, Texas, USA).

### Ethics

All parents signed a written consent form to participate in the PACT study. The Regional Committee for Medical Research Ethics and the Norwegian Data Inspectorate Board approved the study (Ref 120–2000) (Ref 2003/953-3 KBE/-).

The PACT study has been registered in Current Controlled Trials database (ISRCTN28090297).

## Results

By March 31^st^ of 2005, 4780 parents had completed the baseline questionnaires at their child’s second year of age, and by April of 2009, 2192 (46%) of these had also returned the health questionnaire when their children turned 6. Comparison of the baseline characteristics of the children with follow-up data at 6 years (n=2192) and children with no follow-up data (n=2588) are shown in Table 1. Apart from more “smoking mothers” and fewer “homeowners” among children with no follow-up data at 6 years, compared to children with follow-up data at 6 years, the two groups were comparable at baseline. In addition, the mothers of children with follow-up data were slightly older at delivery (mean age 29.8, ± 4.50 SD vs. 28.9, ± 4.79 SD). Among children with follow-up data at 6 years, the prevalence of eczema, asthma and wheezing at 2 years was 17.8% (95% CI 16.2-19.5), 6.5% (95% CI 5.5-7.6) and 25.3% (95% CI 23.5-27.2), respectively. The corresponding reported prevalences for children with no follow-up data showed only small and insignificant differences from these numbers (Table 1).

**Table 1:** Baseline characteristics and prevalence of allergy-related diseases at 2 years of age

	**Children with follow-up data at 6 years (N=2192)**^**a**^			**Children without follow-up data at 6 years (N=2588)**^**a**^		
	**n/N**	**%**	**95% CI**	**n/N**	**%**	**95% CI**
Characteristics						
Male sex	1080/2192	49.3	47.2-51.4	1309/2586	50.6	48.7-52.6
Sibling(s)	1531/2160	70.9	68.9-72.8	1734/2554	67.9	66.0-69.7
Atopy in the family	1474/2157	68.3	66.3-70.3	1733/2543	68.1	66.3-70.0
Breastfed ≥ 3 months	1862/1981	94.0	92.9-95.0	2191/2334	93.9	92.8-94.8
Ever antibiotics	1008/2181	46.2	44.1-48.3	1188/2578	46.1	44.1-48.0
Dog at home	163/2002	8.1	7.0-9.4	202/2364	8.5	7.4-9.7
Cat at home	158/2002	7.9	6.7-9.2	239/2364	10.1	8.9-11.4
Homeowner	1973/2159	91.4	90.1-92.5	2145/2552	84.1	82.6-85.5
Smoking mother	381/2124	17.9	16.3-19.6	548/2503	21.9	20.3-23.6
Smoking father	346/1944	17.8	16.1-19.6	461/2244	20.5	18.9-22.3
Allergy related diseases^b^						
Eczema	386/2169	17.8	16.2-19.5	402/2554	15.7	14.3-17.2
Doctor-diagnosed asthma	143/2191	6.5	5.5-7.6	194/2587	7.5	6.5-8.6
Wheezing	554/2189	25.3	23.5-27.2	701/2585	27.1	25.4-28.9
Rhino-conjunctivitis	119/2182	5.4	4.5-6.5	160/2564	6.2	5.3-7.2
Allergy test performed	462/2185	21.1	19.4-22.9	543/2569	21.1	19.6-22.8
Sensitisation (any)	223/1973	11.3	9.9-12.8	272/2323	11.7	10.4-13.1

^a^Numbers of missing data varies for different variables.

^b^Estimates of ever having the disease.

In Table 2, we compared the estimated prevalence of allergy-related diseases at 2 years of age among those with available data at baseline with the estimated prevalence using multiple imputations; no substantial differences were found. We also compared the estimated prevalence of allergy-related diseases among 6-year-olds with follow-up data to the estimated prevalence found using multiple imputations. The prevalence of asthma and rhino-conjunctivitis at 6 years of age tended to be higher using multiple imputation, but the differences were small (Table 3).

**Table 2:** Prevalence of allergy-related diseases and sensitisation in children at 2 years of age

	**Baseline**			**Multiple imputation (N=4780)**	
	**n**	**%**	**95% CI**	**%**	**95% CI**
Eczema (ever)	788/4723	16.7	15.6-17.8	17.4	16.3-18.5
Asthma (ever)	337/4778	7.1	6.3-7.8	7.1	6.3-7.8
Wheezing (ever)	1255/4774	26.3	25.0-27.6	27.7	26.4-29.0
Sensitised^a^	―	―	―	21.4	16.1-26.7

^a^Only children in the subsample were allergy tested.

**Table 3:** Prevalence of allergy-related diseases in children at 6 years of age

	**Children with follow-up data**			**Multiple imputation(N=4780)**	
	**n**	**%**	**95% CI**	**%**	**95% CI**
Eczema (current)	295/2171	13.6	12.2-15.1	14.0	12.5-15.5
Asthma (current)	121/2192	5.5	4.6-6.6	8.0	6.6-9.3
Rhino-conjunctivitis (ever)	255/2178	11.7	10.4-13.1	14.7	12.9-16.5

More than one-half (56%) of the children with a history of eczema at 2 years did not report current eczema at age 6 years (Table 4). Approximately 42% of those with eczema at age 6 years had their first appearance of eczema after 2 years of age.

**Table 4:** Prevalence of allergy-related diseases reported at 2 and 6 years by eczema-status and sex

	**Reported at 2 years**						**Reported at 6 years**								
	**Asthma**			**Wheezing**			**Eczema**			**Asthma**			**Rhino-conjunctivitis**		
	**n**	**%**	**95% CI**	**n**	**%**	**95% CI**	**n**	**%**	**95% CI**	**n**	**%**	**95% CI**	**n**	**%**	**95% CI**
Reported eczema 2 yrs															
Total (n=386)	39	10.1	7.3-13.6	134	34.7	30.0-39.7	170	44.0	39.0-49.2	41^a^	10.6	7.7-14.1	105	27.2	22.8-31.9
Boys (n=200)	27	13.5	9.1-19.0	76	38.0	31.2-45.1	81	40.5	33.6-47.7	24	12.0	7.8-17.3	57	28.5	22.4-35.3
Girls (n=186)	12	6.4	3.4-11.0	58	31.2	24.6-38.4	89	47.8	40.5-55.3	17	9.1	5.4-14.2	48	25.8	19.7-32.7
Reported no eczema 2 yrs															
Total (n=1783)	102	5.7	4.7-6.9	414	23.2	21.3-25.3	123	6.9	5.8-8.2	79^b^	4.4	3.5-5.5	148	8.3	7.1-9.7
Boys (n=863)	65	7.5	5.9-9.5	222	25.7	22.8-28.8	49	5.7	4.2-7.4	53	6.1	4.6-8.0	88	10.2	8.3-12.4
Girls (n= 920)	37	4.0	2.8-5.5	192	20.9	18.3-23.6	74	8.0	6.4-10.0	26	2.8	1.9-4.1	60	6.5	5.0-8.3

^a^In children with eczema at 2 years: 22/41 children reporting asthma at 6 years also reported asthma at 2 years.

^b^In children without eczema at 2 years: 42/79 children reporting asthma at 6 years also reported asthma at 2 years.

No substantial sex differences were found in the prevalence of eczema at 2 years. Overall, however, boys reported significantly more asthma than girls both at 2 years (5.4% vs. 8.7%, p<0.001) and at 6 years (6.4% vs. 11.2%, p<0.001).

**Figure 1:**
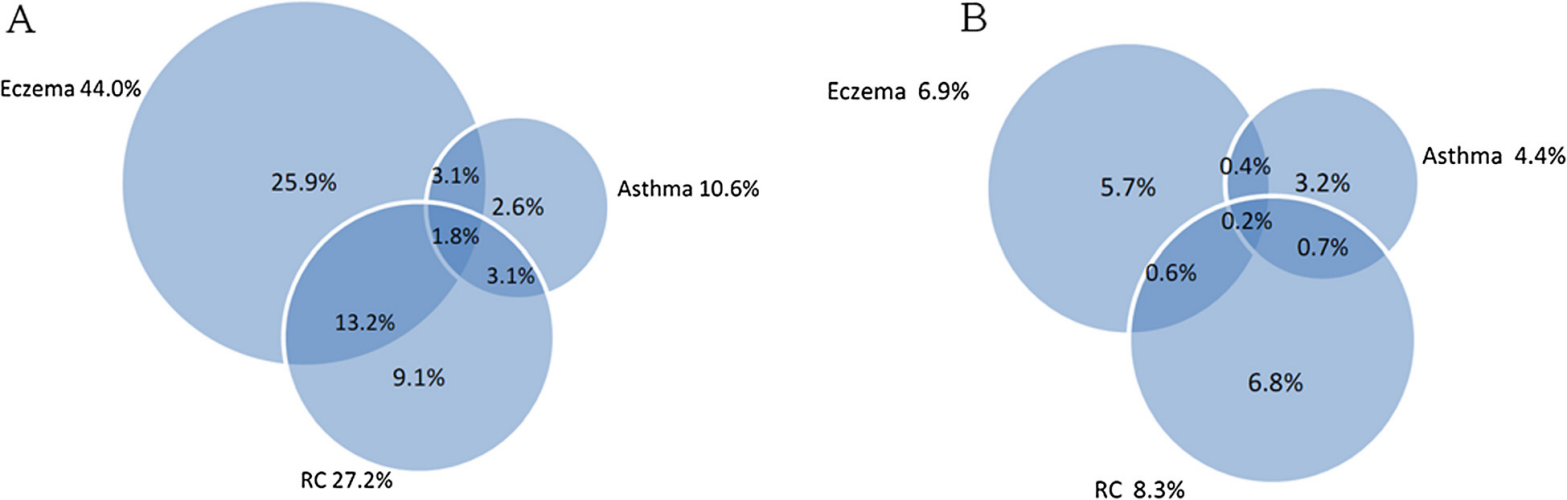
Co-existence of allergy-related diseases reported at age 6 years among children with (A) and without (B) eczema at 2 years of age.

Children with eczema at 2 years reported more asthma at both 2 and 6 years compared to those without eczema. The co-existence of eczema and rhino-conjunctivitis, eczema and asthma and asthma and rhino-conjunctivitis at 6 years of age among children with eczema at 2 years was 15%, 4.9% and 4.9%, respectively. The corresponding numbers for those without eczema at 2 years were all less than 1% (Figure 1).

Among children with follow-up data at 6 years (complete case analysis), the adjusted odds ratio between eczema at 2 years and current asthma at 6 years was 1.95 (95% CI 1.07-3.54) (Table 5). When the age of onset for eczema was included in the model, an onset before four months of age (first tertile) was significantly associated with asthma at 6 years, adjusted OR= 4.51 (95% CI 1.73-11.72) (Table 6). We found no basis for a multiplicative interaction between eczema and sex (p=0.83) or eczema and wheezing (p=0.13) for risk of asthma at 6 years.

**Table 5:** Adjusted associations between reported eczema at age 2 years and current asthma at 6 years

	**Complete case**^**b**^**(N=2192)**^**a**^		**Multiple imputation**^**b**^**(N=4780)**		**Multiple imputation**^**c**^**(N=4780)**	
	**OR**	**95% CI**	**OR**	**95% CI**	**OR**	**95% CI**
Eczema	1.95	1.07-3.54	1.92	1.21-3.04	1.80	1.10-2.96
Male sex	1.70	0.96-3.00	1.20	0.84-1.71	1.19	0.83-1.71
Atopy in family	2.30	1.06-4.96	1.84	1.06-3.20	1.74	0.99-3.05
Wheezing	3.61	2.06-6.31	2.71	1.70-4.32	2.67	1.65-4.32
Sensitisation (any)	―	―	―	―	2.39	0.95-6.02

^a^Numbers of missing data varies for different variables.

^b^Adjusted for homeowner, smoking mother, male sex, atopy in family and wheezing.

^c^Adjusted for ^b^ plus sensitisation.

**Table 6:** Adjusted associations between the eczema age of onset at 2 years and current asthma at 6 years

	**Complete case**^**b**^**(N=2192)**^**a**^		**Multiple imputation**^**b**^**(N=4780)**		**Multiple imputation**^**c**^**(N=4780)**	
	**OR**	**95% CI**	**OR**	**95% CI**	**OR**	**95% CI**
Age of eczema onset in tertiles^d^						
0-3 months	4.51	1.73-11.72	2.18	1.03-4.61	1.98	0.91-4.28
4-12 months	1.21	0.27-5.35	1.40	0.61-3.19	1.33	0.58-3.04
≥13 months	2.94	0.82-10.53	2.11	0.72-6.16	2.05	0.69-6.07
Sex	1.49	0.81-2.74	1.29	0.83-2.00	1.27	0.81-2.01
Atopy in family	2.46	1.08-5.62	1.81	0.99-3.31	1.73	0.92-3.23
Wheezing	4.98	2.72-9.13	2.62	1.55-4.43	2.59	1.52-4.41
Sensitisation (any)	―	―	―	―	2.19	0.77-6.20

^a^Numbers of missing data varies for different variables.

^b^Adjusted for homeowner, smoking mother, male sex, atopy in family and wheeze.

^c^Adjusted for ^b^ plus sensitisation.

^d^Reference group: children with no eczema during the first2 years of life.

The analysis using multiple imputations provided nearly the same associations as the complete case analysis. When information on sensitisation was included in the model, no substantial change in the association between eczema and asthma was found (Table 5). We repeated the analysis after removing all children who reported doctor diagnosed asthma (ever) at 2 years and children with wheezing (ever) at 2 years. The association between eczema at 2 years and asthma at 6 years was still significant with OR=2.06 (95% CI 1.09-3.90) (data not shown).

In the model with age of eczema onset categorised in tertiles, the association between age of eczema onset during the first tertile (before 4 months of age) and current asthma at 6 years did not indicate a clear trend or pattern for the age onset eczema and risk of asthma (Table 6). We found no evidence for a multiplicative interaction between eczema and sensitisation (p=0.70).

Boys had a slightly higher risk than girls of developing asthma, but the differences were not significant.

### The random subsample

At age 2 years, 441 of 720 randomly selected children from the control cohort included since pregnancy were eligible for follow-up. Of these, 390 children (88%) participated in a clinical examination. The prevalence of a history of eczema, doctor-diagnosed asthma and wheezing reported in questionnaires at 2 years was 21.0%, 8.8% and 23.5%, respectively. The prevalence of clinically investigated eczema according to the UKWP diagnostic criteria was 15.9%.

An allergy test, either a SPT or a sIgE, was performed in 91% of the children. Of those with UKWP-Eczema, some 22.4% (95% CI 12.5-35.3.4) had a positive test, while some 14.5% (95% CI 10.7-19.0) of those without UKWP-Eczema tested positive (p=0.7).

## Discussion

In this large, prospective study from the general population, we found a strong association between eczema at age 2 years and asthma at age 6 years. Asthma was reported more often among boys than girls, regardless of eczema status. Four out of ten children with eczema at age 6 had the onset of their eczema after 2 years of age, but the co-existence of allergy-related diseases reported at 6 years was higher among children with an onset of eczema before 2 years of age. More than one-half of the children with eczema during the first two years of life no longer had eczema when they turned 6 years.

This comprehensive study was conducted in the primary health care setting. However, as only close to half of the included children had follow-up data at 6 years, there is a possibility of selection bias [16]. The design of the PACT study is that of multiple, yearly, cross-sectional cohorts of children consecutively included from the year 2000. During the inclusion period, we have observed a decline in the number of participants over time. However, a non-participant study carried out among 391 parents who consecutively attended maternal postnatal care revealed no substantial differences in age, education, familial allergy-related diseases and smoking behaviour when comparing participants and non-participants [16]. This trend is also supported by the baseline data and clinical characteristics among those children with follow-up data at 6 years compared with children with no follow-up data at 6 years. Apart from socioeconomic differences, indicated by fewer homeowners and more smoking mothers among children without available data at 6 years, only minor differences were found. This socioeconomic difference between participants and non-participants is a well-known challenge in population-based studies, and has also been reported by others [7,18]. The low inclusion rate is most likely due to low inclusion activity among GPs, health visitors and midwives and is not likely a consequence of self-selection among parents and children.

Another possible limitation to the study is the use of questionnaires and parental reporting without a clinical verification for some of the diagnoses. The questionnaires are from the ISAAC study, and for the 2-year-olds, we adapted them to fit with this particular age-group. We have validated the eczema diagnoses among the 2-year-olds against the UKWP diagnostic criteria in another study [14] and the results indicate that the prevalence of eczema in this age-group may be overestimated when self-reported. The reliability of some of the questions regarding health has, however, been tested, and reported doctor-diagnosed asthma and the information obtained from medical records showed excellent agreement [19]. In addition, mother-reported use of anti-asthmatic medications during the previous year among 7-year-old children has been shown to be highly valid [20]. The questions on rhino-conjunctivitis have, to the best of our knowledge, not been validated for 2-year-olds, and due to the high rate of infections with symptoms resembling rhino-conjunctivitis in this age group, the prevalence of rhino-conjunctivitis reported at 2 years must be interpreted with caution.

In addition to the early manifestation of eczema, it is commonly believed that the severity of eczema, male sex, early wheezing, heredity and sensitisation are possible risk factors for the development of childhood asthma. We used information from the clinical investigative subsample, in which 91% of the children were tested either by SPT or blood sample, regardless of the presence of symptoms, to conduct multiple imputations on missing sensitisation data. In doing so, the sensitisation estimate is less precise. Another limitation regarding sensitisation is the fact that we have only tested the children once, at the age of 2 years. We might therefore have missed the children with transient sensitisation before this age. In addition, there is also a possibility that children are sensitised for allergens not tested for, such as wheat and soybean.

The strength of this study is the large number of participants, the unselected population, the prospective design, the random clinical subsample at baseline and the validation of several of the questions.

The prevalence of allergy-related diseases among 6-year-olds has been widely documented through the ISAAC study [21]. The 12-month prevalence for eczema in phase III of the study in Sweden, Germany and the UK was 19.5%, 7.9% and 16.0%, respectively. Our finding of 13.6% is in between the prevalence reported in Germany and the U.K. but, surprisingly, is much lower than in Sweden. The estimated asthma prevalence of 5.5% is lower than in all three countries (10.2%, 12.8% and 20.9%, respectively), whereas the prevalence of rhinitis in our study is somewhat higher. The prevalence of eczema, asthma and rhino-conjunctivitis in our cohort of 6-year-olds are, however, well in line with the prevalence found in a Danish birth cohort among children of the same age [22].

Whether eczema is a true risk factor for asthma and rhino-conjunctivitis has been debated, and the relationship between the different allergy-related disorders is unclear. A population-based, longitudinal birth cohort study from the U.K. found that impairment in the skin barrier due to mutations in the FLG gene was associated with an increased risk of eczema, both atopic and non-atopic [13]. In addition, patients with these mutations had more persistent eczema. FLG mutations were also associated with an increased risk of asthma, but only when co-existing with eczema [9,13]. Suggestions were made by the authors that the sub-stratification of eczema based on FLG status could identify those children who might benefit from early therapeutic intervention. We did not, however, have the option of FLG testing for the children participating in this study.

We found that only twenty percent of children with eczema at 2 years were sensitised, whereas others have found sensitisation among children in this age group to be approximately one-third [23]. It has been hypothesised that the course of the disease is different for those with sensitisation as opposed to those without sensitisation, and that rather than a progressive atopic march, there are different phenotypes of eczema leading to asthma [3,24]. Others have found that FLG mutations with co-existing eczema increase the risk for asthma and hay-fever without an obligate presence of concurrent sensitisation [13]. Our results show an increased risk of asthma at age 6 years among children with eczema at 2 years compared to children without eczema at 2 years, regardless of sensitisation.

## Conclusions

Although most cases of eczema in primary health care are mild to moderate, the findings from this study support the hypothesis of an atopic march in the general population.

## Abbreviations

CI: Confidence Interval; FLG: Filaggrin; IgE: Immunoglobulin E; ISAAC: International Study of Asthma and Allergies in Childhood; MI: Multiple Imputations; OR: Odds Ratio; PACT: Prevention of Allergy among Children in Trondheim; SCORAD: Scoring Atopic Dermatitis; sIgE: Specific Immunoglobulin E; SPT: Skin Prick Test; UKWP: United Kingdom Working Party.

## Competing interests

The authors declare that they have no competing interests.

## Authors’ contributions

MS participated in the design of the study, performed the statistical analysis and drafted the manuscript. TØ, OS and RJ conceived of the study, participated in its design and co-ordination and helped draft the manuscript. PRR performed the statistical analyses and helped draft the manuscript. CKD helped with the statistical analyses and helped draft the manuscript. TLH helped draft the manuscript. All authors read and approved the final manuscript.

## Pre-publication history

The pre-publication history for this paper can be accessed here:

http://www.biomedcentral.com/1471-2431/12/168/prepub
